# Role of Copper Homeostasis and Cuproptosis in Cardiovascular Disease: Molecular Insights and Metabolic Perspectives

**DOI:** 10.7150/ijbs.126580

**Published:** 2026-02-18

**Authors:** Xianzhe Yu, Dou Yuan, Yabo Wang, Leibo Wang, Lingling Zhu, Qi An, Yunfei Ling

**Affiliations:** 1 Department of Cardiovascular Surgery, West China Hospital of Sichuan University, No. 37 GuoXue Xiang, Chengdu, Sichuan 610041, China.; 2 Department of Cardiovascular Surgery, Chengdu Shang Jin Nan Fu Hospital, West China Hospital of Sichuan University, Chengdu, Sichuan 610041, China.; 3 Institute of Human Genetics, Jena University Hospital, Jena 07747, Germany.; 4 Department of Medical Oncology, West China Hospital of Sichuan University, No. 37 GuoXue Xiang, Chengdu, Sichuan 610041, China.; 5 Lung Cancer Center/Lung Cancer Institute, West China Hospital of Sichuan University, No. 37 GuoXue Xiang, Chengdu, Sichuan 610041, China.

**Keywords:** copper, cuproptosis, copper homeostasis, cardiovascular disease, mitochondrial dysfunction

## Abstract

Copper is an essential trace element; however, its homeostasis is frequently disrupted in cardiovascular diseases, which are a leading cause of mortality worldwide. The recent discovery of cuproptosis—a copper-dependent form of regulated cell death (RCD)—has provided a crucial mechanistic link between this imbalance and cardiomyocyte loss. In this review, we synthesize the current understanding of how dysregulated copper metabolism and cuproptosis drive the pathogenesis of major cardiovascular conditions, including myocardial ischemia/reperfusion (I/R) injury, anthracycline-induced cardiotoxicity, atherosclerosis, diabetic cardiomyopathy (DCM), and sepsis-induced cardiac dysfunction, through pathways such as mitochondrial dysfunction, oxidative stress, and inflammation. We further evaluated emerging therapeutic strategies that target copper homeostasis—including chelators, chaperone inhibitors, and ionophores—and critically analyzed the translational challenges they face, such as off-target effects and preclinical model limitations. Advancing our knowledge of cardiac copper biology holds significant promise for the development of novel and precise therapeutic approaches for cardiovascular diseases.

## Introduction

Cell death is a critical driver of cardiovascular disease pathogenesis, contributing to tissue damage, dysfunction, and inflammation 1. Among the various regulated cell death (RCD) pathways, a novel form—cuproptosis—has recently emerged, uniquely triggered by disruptions in copper metabolism 2. Mechanistically distinct from other forms of cell death, cuproptosis is characterized by the direct binding of copper to lipoylated enzymes in the mitochondrial tricarboxylic acid (TCA) cycle, leading to proteotoxic stress and subsequent cell death, independent of classical apoptotic signaling 3.

Copper, an essential trace element, acts as a cofactor for numerous metabolic enzymes and is vital for physiological processes, including antioxidant defense, mitochondrial respiration, and energy metabolism 4. Cellular function relies on a tightly regulated copper metabolic network that maintains copper concentrations within a narrow range. Copper deficiency impairs copper-dependent enzymes, whereas copper overload promotes excessive reactive oxygen species (ROS) generation, leading to oxidative damage to lipids, proteins, and DNA 5, 6. This oxidative stress can activate classical apoptotic pathways, a process termed copper-induced apoptosis, which is mediated by DNA/membrane damage and the caspase/Bcl-2/p53 pathway and can be inhibited by caspase inhibitors 7. In contrast, cuproptosis represents a separate copper-dependent pathway in which excess copper directly targets mitochondrial metabolism, inducing cell death through aggregated lipoylated proteins and iron-sulfur (Fe-S) cluster loss, independent of apoptotic signaling 3, 8. Copper-induced oxidative stress, although a key modulator of cellular damage, is not a direct cell death pathway. It is a cellular stress state resulting from copper-driven ROS accumulation, which can facilitate apoptosis or other death modalities, such as ferroptosis, and can be alleviated by antioxidants 9, 10.

Given that cellular function depends critically on precise copper homeostasis, any disturbance in this balance can have severe pathological consequences 11. As a novel RCD modality involving multiple signaling and metabolic pathways, cuproptosis has been implicated in various cardiovascular conditions, including cardiomyopathy, heart failure (HF), and myocardial injury 12. Therefore, this review aims to summarize the regulation of copper homeostasis in cardiomyocytes, explore cuproptosis-related targets in cardiovascular diseases, and discuss the translational potential of therapies targeting this pathway.

## Molecular and Metabolic Drivers of Cuproptosis

Cells undergoing cuproptosis exhibit unique biochemical, genetic, metabolic, and morphological features that distinguish them from other recognized forms of cell death, such as apoptosis, pyroptosis, necroptosis, and ferroptosis 13, 14 **(Table 1)**. Apoptosis is a caspase-dependent process activated by intrinsic (e.g., mitochondrial outer membrane permeabilization) or extrinsic (e.g., death receptor activation) stimuli. Key cardiac triggers include hypoxia, oxidative stress, and neurohormonal overload 15. Its morphological hallmarks include cell shrinkage, chromatin condensation, and formation of apoptotic bodies 15. Ferroptosis is driven by iron-dependent lipid peroxidation, primarily initiated by glutathione (GSH) depletion or inactivation of GSH peroxidase 4 (GPX4). It is a key contributor to myocardial ischemia/reperfusion (I/R) injury 16. Morphologically, it is associated with distinct mitochondrial changes, such as mitochondrial reticulum shrinkage and fragmentation 3. Although cuproptosis can intersect with pathways such as apoptosis and ferroptosis, its initiation and execution are uniquely co-regulated by interconnected metabolic pathways involving copper, GSH, and lipids, which are particularly critical in cardiomyocytes 17. Given these distinct mechanisms, cuproptosis has been implicated as a contributing factor in the pathogenesis of diverse cardiovascular diseases, including dilated cardiomyopathy, HF, and atherosclerosis 12.

### Heart Copper Metabolism and Cuproptosis

#### Regulation of Copper Homeostasis in the Cardiovascular System

Copper homeostasis is a tightly regulated process that is essential for cardiovascular health and balances systemic absorption, distribution, utilization, and excretion. This equilibrium is particularly critical in high-energy-demand tissues, such as the heart, where copper serves as an indispensable cofactor for key metabolic enzymes and mitochondrial function 18, 19. Intracellularly, copper buffering by molecules such as GSH and metallothionein (MT) prevents ROS generation via Fenton reactions 20. Specific chaperones then deliver copper to target proteins; for instance, the copper chaperone for superoxide dismutase (CCS) activates Cu/Zn superoxide dismutase 1 (SOD1), which is a key antioxidant enzyme in vascular cells 21. Notably, CCS expression is regulated by copper levels, increasing during deficiency and degrading under excess conditions 22.

The cardiovascular roles of copper are multifaceted. As the catalytic core of cytochrome c oxidase (complex IV, CCO), it is fundamental to mitochondrial adenosine triphosphate (ATP) production 23. It also activates other crucial enzymes, including extracellular SOD3, ferroxidase ceruloplasmin (CP) (vital for iron metabolism), and lysyl oxidase (LOX) 24. LOX crosslinks collagen and elastin, providing structural integrity and elasticity to the heart and vasculature 25. The critical dependence on copper is underscored by pathologies arising from its dysregulation. For example, copper restriction impairs CCO assembly and function, leading to reduced mitochondrial ATP production and oxygen consumption 26. Beyond metabolism and structure, copper promotes angiogenesis by stabilizing hypoxia-inducible factor-1α (HIF-1α) via transporters such as Copper Transporter 1 (CTR1; Solute Carrier Family 31 Member A1, SLC31A1) and ATPase Copper Transporting Alpha (ATP7A), thereby enhancing pro-angiogenic gene expression 27, 28.

**Figure 1:**
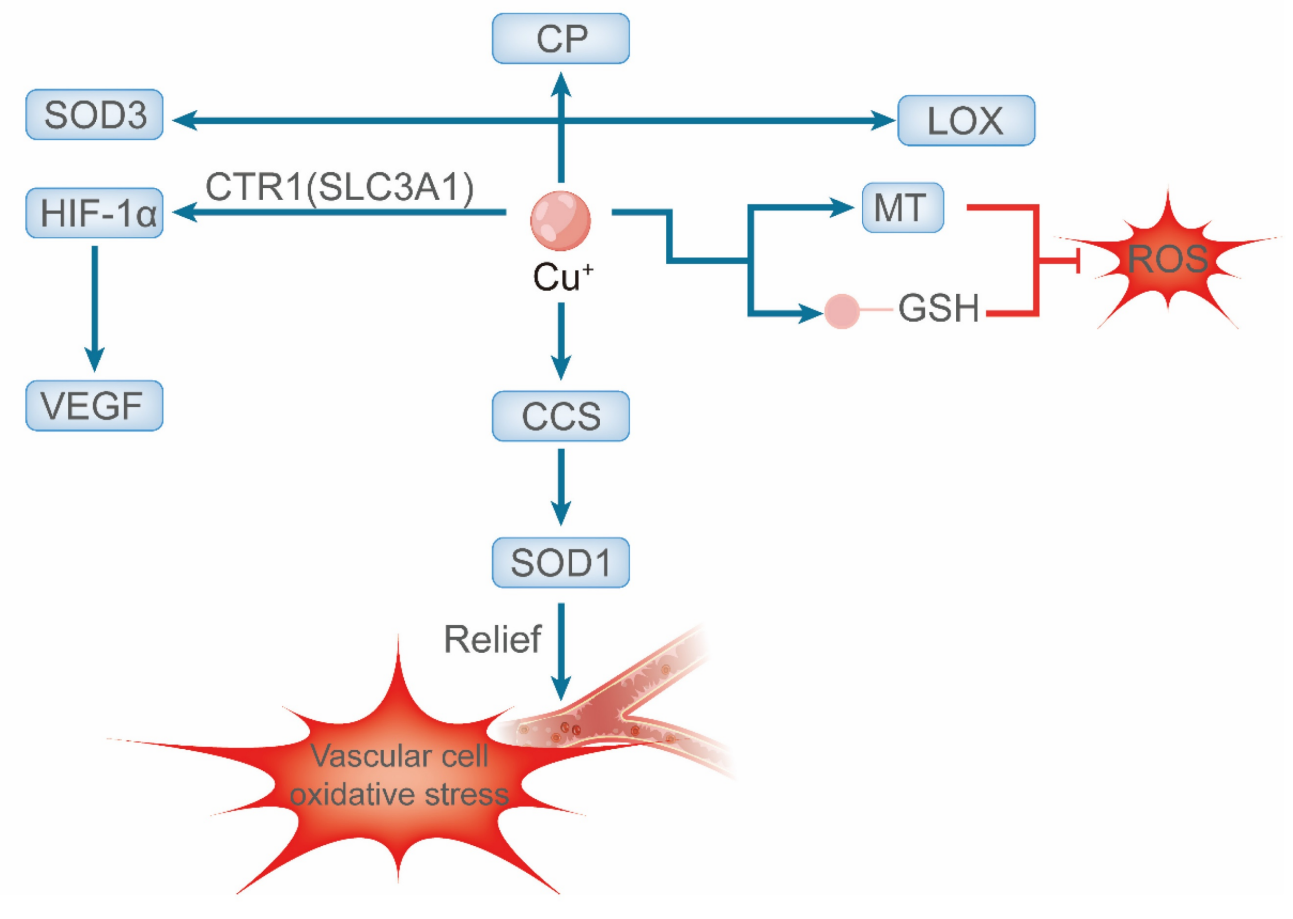
Schematic representation of copper-mediated regulation of vascular redox homeostasis. Intracellularly, Cu⁺ acts as an essential cofactor that activates secretory enzymes (e.g., CP and LOX) is delivered to SOD1 via CCS to combat superoxide and stabilizes HIF-1α to promote VEGF expression. Cellular redox balance is maintained by copper buffers (MT) and antioxidants (GSH).

Given these essential functions, serum copper levels are closely associated with cardiometabolic risk factors, including dyslipidemia, type 2 diabetes, and obesity, and are regarded as predictive indicators of cardiovascular disease risk 29. In summary, disruptions in copper homeostasis—whether arising from deficiency, excess, or dysfunction of molecular chaperones and transporters—are fundamentally implicated in the pathogenesis of various cardiovascular diseases 30 **(Figure 1)**.

#### Copper Homeostasis Imbalance and Cardiovascular Disease

Copper overload induces cardiovascular injury through multiple interconnected mechanisms. Systemically, excess copper catalyzes Fenton-like reactions, generating excessive ROS that cause oxidative damage to lipids, proteins, and DNA 31. It concurrently impairs antioxidant defenses (e.g., by inhibiting catalase) 32 and promotes inflammation by elevating pro-inflammatory cytokine levels and reducing nitric oxide (NO) bioavailability 33. At the cellular level, these insults trigger distinct pathogenic pathways. In endothelial cells (ECs), copper overload disrupts mitochondrial dynamics, increases fission and ROS production, and ultimately leads to cell death 34 In cardiomyocytes, copper promotes hypertrophy and inflammation 35, disrupts fatty acid and lipid metabolism, upregulates autophagy-associated proteins and genes, and impedes calcium ion uptake by the sarcoplasmic reticulum, thereby inhibiting myocardial contractility 36. Chronic copper overload is a potent driver of pathological cardiac remodeling, primarily by inducing sustained oxidative stress. The accumulation of copper catalyzes excessive ROS production, which directly damages mitochondrial integrity and function and activates pro-fibrotic signaling pathways—most notably transforming growth factor-beta (TGF-β) 37. These structural and cellular alterations culminate in adverse outcomes. The resulting myocardial fibrosis disrupts the normal electrical conduction system of the heart, creating a substrate for electrophysiological instability. This, combined with the accompanying hypertrophic and neurohormonal changes, significantly increases the susceptibility to severe arrhythmias, including ventricular tachycardia and atrial fibrillation 38, 39. Elevated serum copper levels, often observed in aging and diabetes, are both a driver and a biomarker of vascular damage, forming a vicious cycle that accelerates vascular aging and increases the risk of cardiovascular disease 40, 41. Recent bioinformatic studies have further underscored its clinical relevance by highlighting a pronounced cuproptosis signature in myocardial infarction, involving key regulators such as SLC31A1 and ferredoxin-1 (FDX1) 42.

In contrast, copper deficiency promotes cardiovascular pathology through distinct mechanisms, primarily mitochondrial dysfunction and impaired structural integrity 43. Deficiency disrupts the assembly and function of CCO by impairing copper-delivery proteins (e.g., COX17, SCO1/SCO2), severely reducing ATP production 44. The resultant energy crisis and diminished SOD activity increase oxidative stress, promote low-density lipoprotein (LDL) oxidation 45, and disrupt calcium homeostasis, leading to diastolic dysfunction and HF 46.

In the myocardium, a reduction in LOX activity disrupts the connective tissue architecture and impairs contractile function 47. Conversely, elevated LOX activity promotes pathological cardiac remodeling by driving excessive collagen crosslinking and deposition. This leads to myocardial fibrosis, increased tissue stiffness, impaired angiogenesis, and ultimately contributes to HF 48, 49. These defects ultimately lead to extensive structural and functional pathological alterations. The heart undergoes concentric hypertrophy, characterized by thickened ventricular walls without cavity enlargement, resembling pressure overload hypertrophy 50.

**Figure 2:**
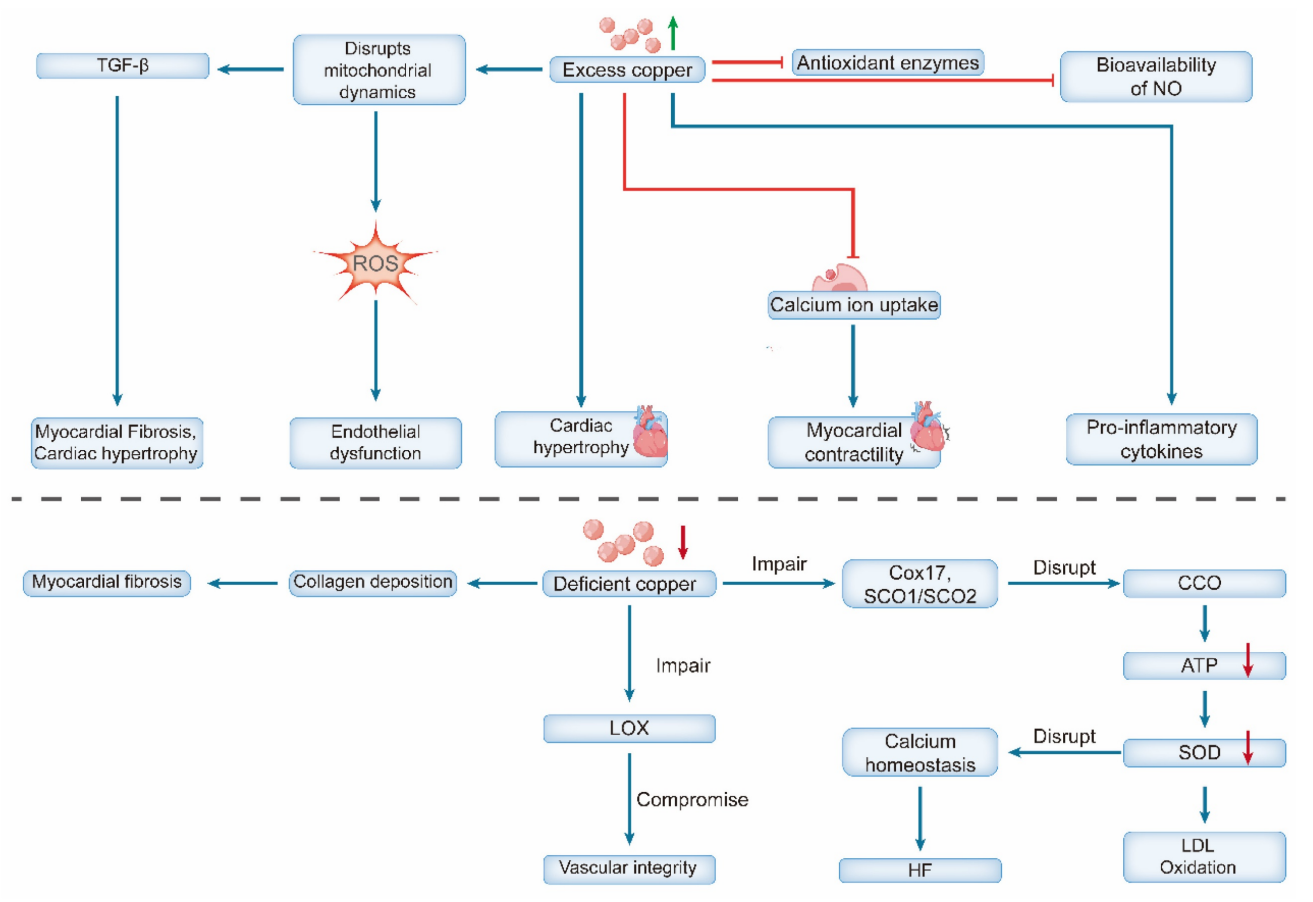
Dual effects of copper dyshomeostasis on cardiovascular pathophysiology. Copper excess triggers oxidative stress (by impairing antioxidant enzymes and NO bioavailability) and disrupts Ca²⁺ homeostasis, leading to endothelial dysfunction, cardiac hypertrophy, and contractile impairment. Copper deficiency compromises cuproenzyme function, causing mitochondrial dysfunction (via impaired CCO), reduced antioxidant defense (via SOD downregulation), and structural defects (via reduced LOX activity leading to fibrosis). Collectively, these changes promote heart failure.

A critical aspect of copper deficiency is that its effects are often reversible. Copper supplementation in both animal models and humans (e.g., patients with SCO2 mutations) can restore CCO activity, reverse hypertrophy and fibrosis, and significantly improve cardiac function 51, 52. This reversibility underscores the crucial and dynamic role of copper in maintaining cardiovascular health **(Figure 2)**.

#### Copper Metabolism in Cuproptosis

Cuproptosis is a unique form of RCD driven by the cytotoxic accumulation of copper, with its upstream regulation and execution being critically dependent on the mitochondrial reductase FDX1 53. It reduces Cu²⁺ to the more reactive Cu⁺ and concurrently supplies electrons for the biosynthesis of the lipoyl moiety on key mitochondrial enzymes, such as Dihydrolipoamide Acetyltransferase (DLAT) 54. This lipoylation creates high-affinity Cu ^+^ binding sites. Subsequent copper binding induces aberrant oligomerization of these proteins, disrupting the TCA cycle and initiating cell death 55.

The FDX1-mediated process culminates in profound proteotoxic stress driven by the aggregation of lipoylated mitochondrial proteins and the concomitant destabilization of Fe-S cluster proteins, which together lead to acute mitochondrial failure 3. The copper ionophore elesclomol exhibits a paradoxical dual role that is critically dependent on the cellular context. In the presence of FDX1 and adequate copper, cuproptosis is induced by delivering extracellular Cu²⁺ into cells 56. Conversely, under conditions of copper deficiency or FDX1 loss, elesclomol shifts to a protective function, restoring mitochondrial respiration by delivering copper to CCO 57. This duality highlights the precise metabolic context required for cuproptosis. The physiological importance of copper import is further underscored by the essential role of the standard importer SLC31A1, as evidenced by reduced cardiac copper levels in SLC31A1-deficient mice 50, 58.

**Figure 3:**
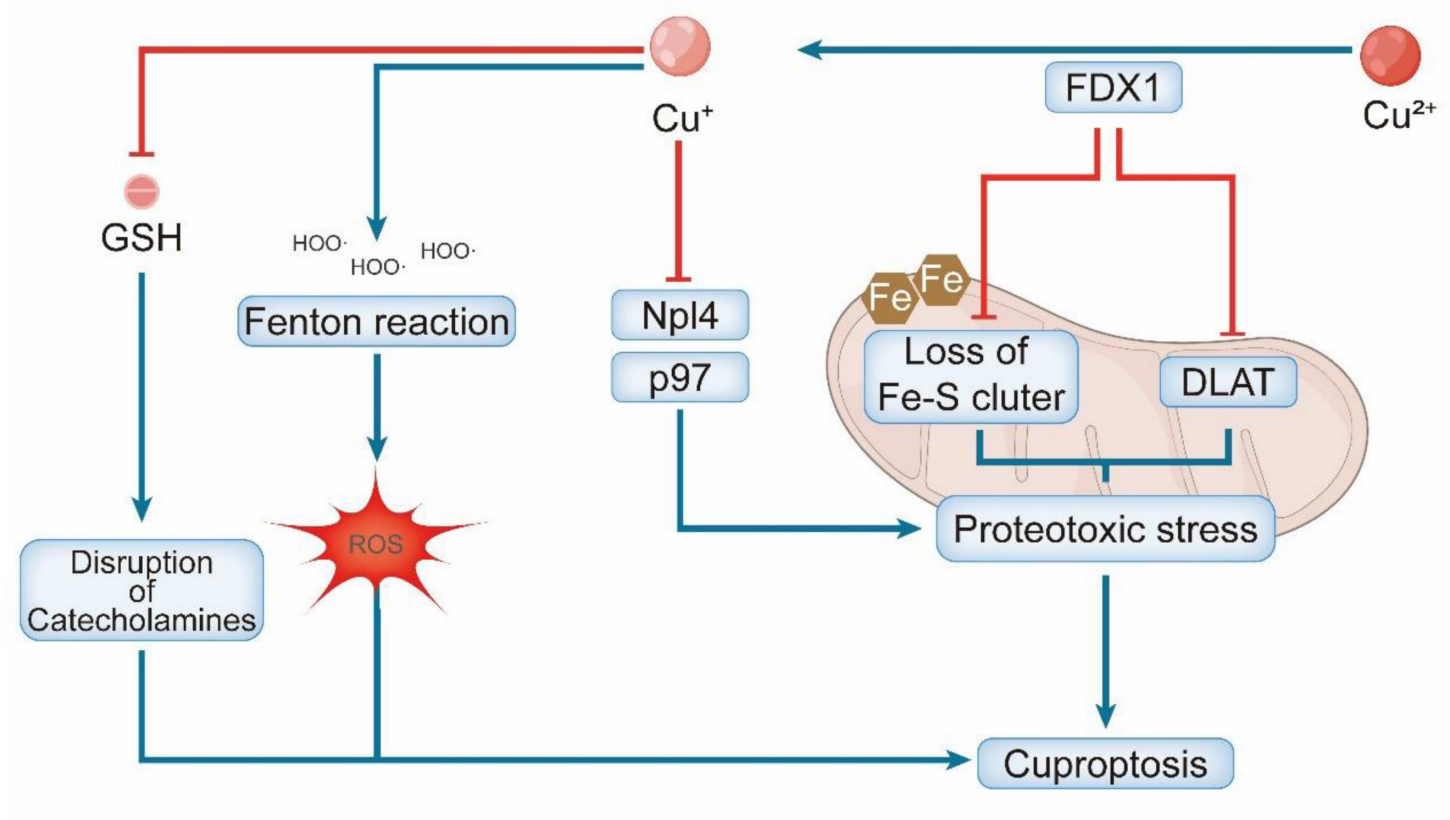
Schematic representation of copper-induced cuproptosis. Excess copper is reduced by FDX1 to generate Cu⁺, which drives cell death via two primary mechanisms: (1) Cu⁺-mediated aggregation of lipoylated TCA cycle proteins (e.g., DLAT) and loss of Fe-S clusters, and (2) Cu⁺-induced disruption of the Npl4/p97 protein quality-control system. Both pathways converge to induce fatal proteotoxic stress in the mitochondria. Elevated Cu⁺ levels also deplete glutathione (GSH) and promote ROS generation, further contributing to cellular toxicity.

Furthermore, cuproptosis is amplified by secondary mechanisms. Copper ions can participate in Fenton-like reactions, generating ROS that cause oxidative damage to DNA and lipids 59. Additionally, copper may inhibit the p97-Npl4 complex, which is essential for protein degradation, thereby exacerbating underlying proteotoxic stress 60. GSH depletion and copper-catalyzed oxidation of catecholamines generate toxic quinones and ROS, which synergistically contribute to cuproptosis 3. These parallel pathways collectively intensify the proteotoxic and oxidative burdens, ultimately leading to irreversible cell death **(Figure 3)**.

#### Crosstalk between Ferroptosis and Cuproptosis

Ferroptosis, an iron-dependent form of RCD driven by GSH depletion and GPX4 inactivation, is potently accelerated by copper through multi-pronged mechanisms that converge on iron-dependent lipid peroxidation 61, 62. Copper promotes ferroptosis primarily by dismantling the core GPX4-centered antioxidant defense system. It depletes the intracellular pool of GSH, which is GPX4's essential cofactor, through the formation of Cu-GSH complexes and catalyzing its oxidation to glutathione disulfide (GSSG) 63, 64. More critically, copper can directly bind to the cysteine residues of GPX4, triggering its autophagic degradation and consequent loss of activity, which impairs the cell's ability to repair lipid peroxidation 65.

Fe²⁺ acts as a key catalyst driving lipid peroxidation. The Fenton reaction generates highly reactive hydroxyl radicals. These radicals directly target polyunsaturated fatty acids within cell membranes, thereby initiating and propagating the chain reaction of lipid peroxidation 66. This pro-ferroptotic effect is significantly amplified by the intricate crosstalk between copper and iron metabolism. Copper modulates post-translational regulation of iron homeostasis, potentially by inhibiting Fe-S cluster biosynthesis, and transcriptionally upregulates key iron uptake genes, such as transferrin receptor 1. These actions collectively lead to an expansion of the intracellular labile iron pool, thereby supplying more catalysts for Fenton reactions and intensifying lipid peroxidation 67, 68. In cardiovascular diseases, the ferroptosis and cuproptosis pathways exhibit significant crosstalk. Copper stress promotes iron-dependent lipid peroxidation, whereas ferroptosis-derived mtROS enhance cuproptosis by accelerating DLAT oligomerization 69. This interplay creates a vicious cycle of cardiomyocyte and vascular cell death, exacerbating cellular injury and pathological remodeling.

The clinical significance of the copper-ferroptosis axis is underscored by bioinformatic analyses revealing the co-dysregulation of ferroptosis and cuproptosis signatures in human diseases 70. The shared dysregulation of key genes (e.g., POR, SLC7A5, and STAT3) in conditions such as sepsis-induced cardiomyopathy highlights the interplay between these metal-dependent death pathways 71. Furthermore, the copper transporter ATP7A has been identified as a novel ferroptosis regulator, as its deficiency downregulates the cystine transporter SLC7A11, impairing GSH synthesis and sensitizing cells to ferroptosis 72. Collectively, these findings illustrate an intricate network in which copper ions modulate cell fate by targeting multiple nodes of the ferroptotic pathway, presenting a promising avenue for therapeutic interventions.

#### Chemotherapy-induced Cardiac Insult: Copper Homeostasis Disruption

Chemotherapy-induced cardiotoxicity encompasses a range of cardiovascular complications, including cardiomyopathy, HF, and arrhythmias. Among the key causative agents, anthracyclines, such as doxorubicin (DOX), are particularly notable, as the severe cardiac damage they induce—characterized by ventricular dilatation, interstitial fibrosis, and progression to HF—significantly limits their clinical utility 73. DOX-induced cardiotoxicity is driven by established mechanisms, such as oxidative stress, and centrally by dysregulated copper homeostasis and cuproptosis. Disruption of this critical ion balance impairs essential cardiac functions, including the maintenance of myocardial structure, electrical conduction, and contractile performance 74, 75.

This detrimental cascade begins with pathological intracellular copper accumulation. Mechanistically, DOX stabilizes the copper importer SLC31A1 (CTR1) by inhibiting its proteasomal degradation and downregulating the copper efflux transporter ATPase Copper Transporting Beta (ATP7B), leading to a net increase in cellular copper 17, 76. The cuproptosis pathway is triggered in this copper-rich environment. DOX upregulates the expression and activity of FDX1, a reductase that converts accumulated Cu²⁺ to the more reactive Cu⁺ 77. Furthermore, DOX upregulates the expression of key mitochondrial enzymes, including DLAT 78. The increased abundance of lipoylated proteins, combined with the elevated labile copper pool induced by DOX, creates a permissive environment that drives cuproptosis. Cu⁺ ions directly bind to the lipoyl groups, inducing aberrant oligomerization of DLAT and other TCA cycle enzymes, promoting the loss of Fe-S cluster proteins. This cascade results in irreversible proteotoxic stress, ultimately leading to cardiomyocyte death 74. The pivotal role of FDX1 in this pathway is underscored by experimental evidence that genetic ablation of FDX1 confers significant protection against DOX-induced cardiotoxicity in mice, preserving the left ventricular ejection fraction 76. The intrinsic oxidative damage mediated by DOX is further amplified by Cu⁺-catalyzed Fenton-like reactions. This compounded oxidative insult exacerbates mitochondrial membrane damage, respiratory chain dysfunction and DNA damage 79, 80.

#### Physiological Copper Metabolism

Copper homeostasis is maintained through a tightly coordinated process involving systemic absorption, distribution, and excretion. Dietary copper absorption occurs primarily in the stomach, duodenum, and small intestine 81. In this process, Cu²⁺ is first reduced to Cu⁺ by metalloreductases, such as duodenal cytochrome B (DCYTB) and six-transmembrane epithelial antigen of the prostate (STEAP). The resulting Cu⁺ is then transported to the apical membrane of intestinal cells via copper importers, including CTR1/SLC31A1, CTR2, and divalent metal transporter 1 (DMT1) 82. Subsequently, the copper-transporting ATPase ATP7A facilitates copper efflux from these cells into the portal circulation, a process that is upregulated during copper deficiency to enhance absorption 83. ATP7A, which mediates dietary copper absorption in the intestine, and ATP7B, which promotes hepatic copper excretion, exert complementary functions that are critical for maintaining systemic copper homeostasis 84.

The liver is the central hub for systemic copper regulation. Dietary copper, which is bound to albumin, enters hepatocytes via the portal vein 85. Within the liver, the closely related ATPase ATP7B performs two critical location-dependent functions: under normal conditions, it loads copper onto CP in the Golgi apparatus for secretion into the bloodstream, and during copper excess, it traffics to the biliary canaliculus to expel excess copper into the bile 86, 87. This biliary pathway is the primary and irreversible route of copper elimination 88. The entire system is dynamic; high copper intake downregulates intestinal absorption and upregulates biliary excretion, whereas deficiency states promote intestinal uptake and reduce biliary loss to conserve copper 89.

**Figure 4:**
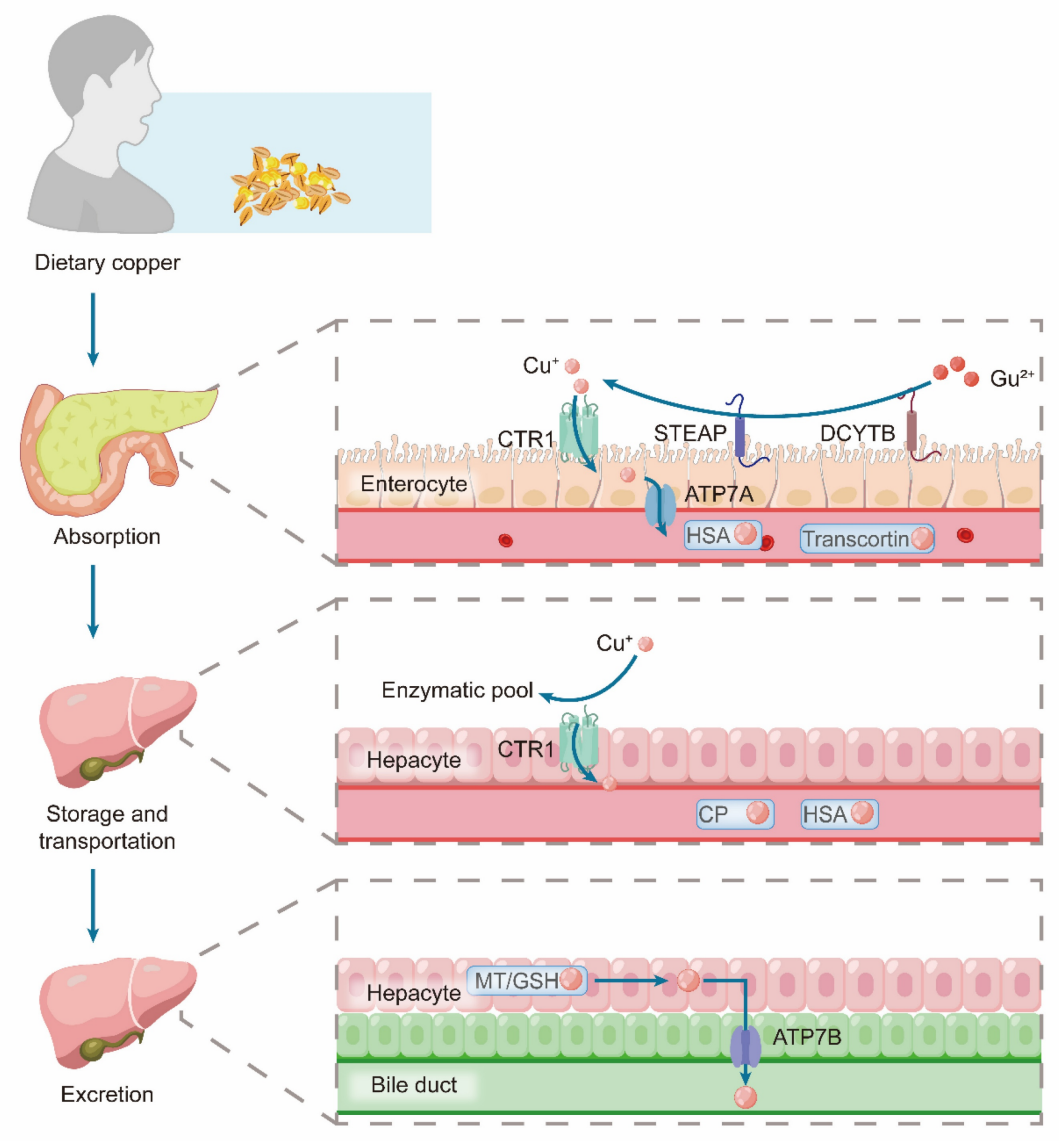
Schematic representation of copper metabolism in the body, including absorption, storage, transport, and excretion. Dietary copper is absorbed by enterocytes, where Cu²⁺ is reduced and transported via proteins such as CTR1, STEAP, DCYTB, and ATP7A, and then carried in circulation by HSA and Transcortin. In hepatocytes, copper is taken up via CTR1 for incorporation into enzymatic pools or is transported by CP and HSA. Excretion occurs through the bile duct, with ATP7B and MT/GSH mediating copper transport into bile.

At the cellular level, a sophisticated chaperone network minimizes cytotoxic free copper levels. Upon entry via CTR1, copper is buffered by GSH or MTs 90. The cytoplasmic chaperone Antioxidant 1 (ATOX1) then distributes copper to the ATP7A and ATP7B transporters in the trans-Golgi network for incorporation into cuproenzymes 91. Under copper overload, these transporters relocate to vesicles or the plasma membrane to mediate the efflux 92. Beyond its cytoplasmic role, ATOX1 can translocate to the nucleus and function as a copper-dependent transcription factor, potentially regulating pathways such as HIF-1α signaling 93** (Figure 4)**.

Additional chaperones target copper to specific organelles. CCS delivers copper to SOD1 in the cytoplasm and intermembrane space, whereas COX17 relays it to the mitochondria 83. Within the mitochondria, copper is passed through secondary chaperones (SCO1, SCO2, and COX11) for incorporation into CCO, which is essential for respiratory function 94. Proteins such as MEMO1 further fine-tune this network by suppressing ATOX1-mediated ROS under excess copper 95. This precise regulation is critical for cardiovascular function. In the vasculature, it stabilizes endothelial NO synthase to preserve NO bioavailability and vasodilatory function 96.

#### Copper Homeostasis Dysregulation and Myocardial Pathological Reprogramming

The progression of cardiovascular diseases, such as myocardial infarction and HF, is driven by core pathological processes, including structural remodeling (e.g., hypertrophy and fibrosis) and metabolic reprogramming of cardiomyocytes toward a fetal-like state 97. A growing body of evidence has identified disrupted copper homeostasis and subsequent activation of cuproptosis as key upstream drivers of this maladaptive process 98. Pathological stressors, such as pressure overload or myocardial infarction, create an inflammatory microenvironment that elevates local copper concentrations, partly due to its release from necrotic cells and upregulated import in immune cells 99. Excess copper exerts a dual pathological effect. First, it directly induces cardiomyocyte loss through cuproptosis, characterized by mitochondrial lipoylated protein aggregation and respiratory chain collapse 100. Second, profound metabolic and oxidative stress from cuproptosis acts as a potent trigger for pathological myocardial reprogramming. It forces a metabolic shift from oxidative phosphorylation to glycolysis and activates established pro-reprogramming signaling pathways, such as YAP/TAZ, which exacerbate inflammatory responses and fibrosis, thereby accelerating adverse ventricular remodeling 49, 101. In vascular smooth muscle cells (VSMCs), it disrupts the TCA cycle and provokes ROS production, leading to cellular hypertrophy and phenotypic switching, which culminates in pathological thickening of the vascular wall 102.

Conversely, copper deficiency contributes to pathology via distinct mechanisms. It impairs the activity of SOD1, exacerbating intracellular oxidative stress and sensitizing cardiomyocytes to damage 12, 103. Furthermore, by reducing CCO activity and compromising oxidative phosphorylation, copper deficiency compels cardiomyocytes to rely more heavily on anaerobic glycolysis, thereby reinforcing the metabolic shift that underpins pathological reprogramming 104. In summary, both excess and deficiency of copper converge to promote myocardial reprogramming and remodeling, highlighting copper homeostasis as a critical nodal point in the pathogenesis of cardiovascular diseases.

### GSH Metabolism and Cuproptosis in the Heart

GSH, a tripeptide composed of glycine, cysteine, and glutamic acid, is a pivotal hub in cellular defense, functioning as both a primary antioxidant and a crucial regulator of copper ion bioavailability 105. Its cardioprotective role is mediated primarily through two synergistic mechanisms: direct copper chelation and maintenance of redox homeostasis 106.

Beyond simple chelation, GSH also acts as a molecular chaperone, facilitating the delivery of Cu⁺ to specific copper chaperones (e.g., ATOX1) and supporting their function, particularly in copper export 107. Crucially, the GSH/Glutaredoxin 1 system maintains the reduced state of cysteine residues within the copper-binding motifs of the transporters ATP7A and ATP7B, which is essential for their copper-transport activity 108.

These dual roles converge to inhibit cuproptosis. Mitochondrial GSH sequesters copper, preventing its binding to lipoylated proteins, such as DLAT, thereby blocking the aberrant protein oligomerization that drives this cell death pathway 109. Consequently, intracellular GSH depletion increases the free copper pool, promotes protein oligomerization, and heightens sensitivity to copper-mediated cell death 110.

Under cardiac stress conditions, such as I/R injury, HF, or drug toxicity, GSH depletion initiates a vicious cycle of damage. The resulting increase in free copper exacerbates oxidative stress and proteotoxicity, further depleting GSH pools and impairing its metal-binding capacity 111, 112. This cycle is amplified by copper's ability to catalyze the oxidation of catecholamines, generating additional toxic quinones and ROS that contribute to cardiotoxicity 113.

Notably, GSH's cardioprotective role extends beyond cuproptosis. As an essential cofactor for GPX4, GSH reduces lipid peroxides and inhibits ferroptosis 114. However, this function can be compromised during copper overload, as Cu²⁺ binding to the cysteine residues of GPX4 promotes its ubiquitination and autophagic degradation, thereby disrupting lipid peroxide metabolism and sensitizing cells to ferroptosis 65. This finding reveals a complex interactive network between copper dyshomeostasis, oxidative stress, and distinct programmed cell death pathways involved in cardiac pathology.

### Lipid Metabolism and Cuproptosis in the Heart

Copper and lipid metabolism exhibit complex bidirectional interactions that critically influence the development and progression of cardiovascular diseases 115. Dysregulation of one pathway often propagates dysfunction in another, establishing a vicious cycle that amplifies cellular injury and ultimately drives disease progression 116.

Copper exerts a profound influence on lipid metabolism, with both deficiency and excess producing distinct but detrimental effects 117. Copper deficiency primarily disrupts lipid homeostasis by impairing the activation of key transcription factors, sterol regulatory element-binding proteins (SREBP-1 and SREBP-2), which regulate fatty acid and cholesterol synthesis 88. This disruption leads to suppressed lipogenesis and failure to maintain normal lipid homeostasis. Furthermore, copper deficiency concurrently promotes triglyceride hydrolysis and enhances fatty acid β-oxidation, creating a state of metabolic imbalance that contributes to pathology 118. These disturbances, compounded by impaired mitochondrial fatty acid oxidation, ultimately contribute to dyslipidemia—characterized by elevated serum triglycerides, LDL cholesterol (LDL-C), and total cholesterol—which accelerates pathological processes such as atherosclerosis and fatty liver disease 119, 120.

**Figure 5:**
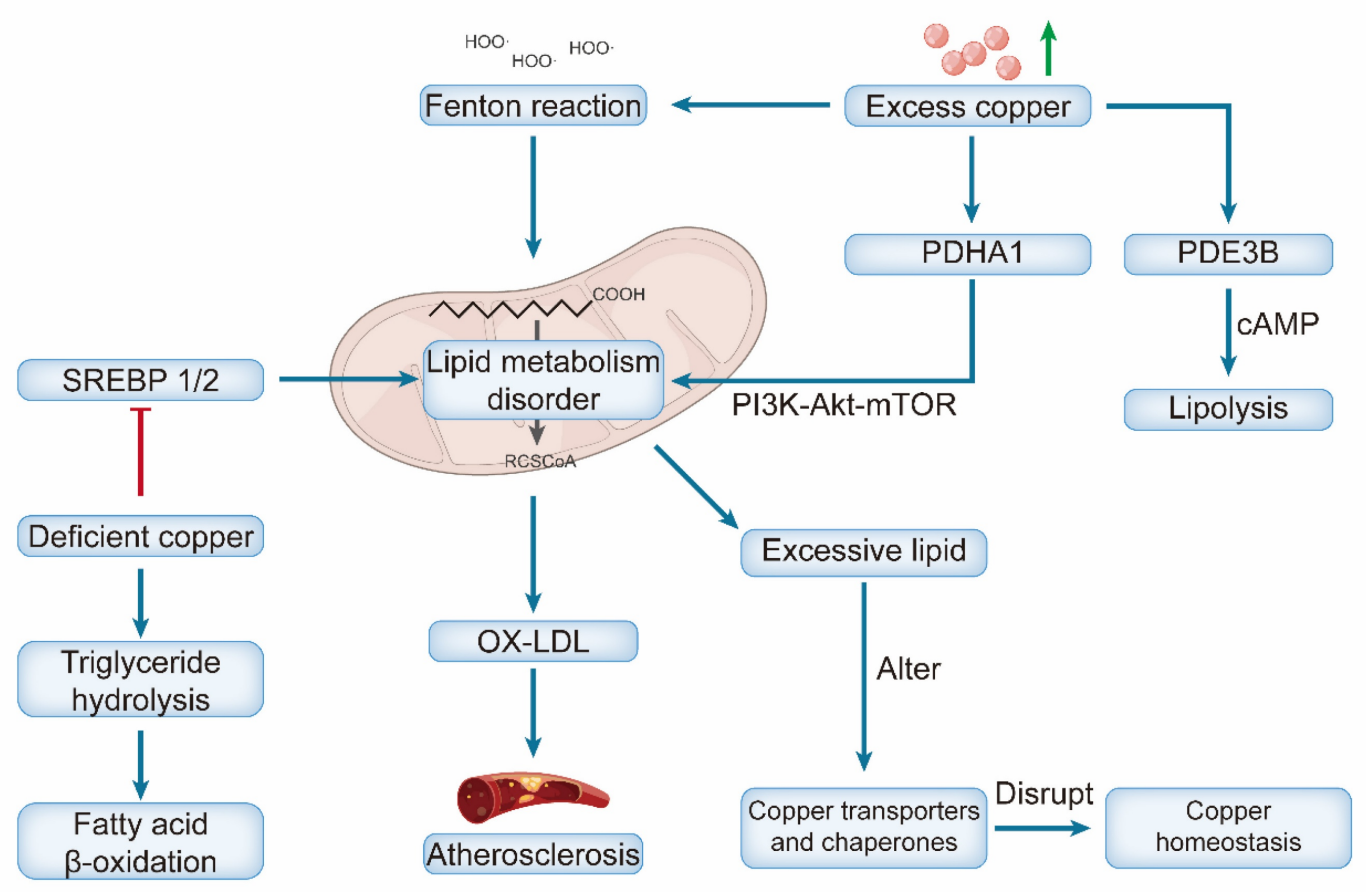
Dual effects of copper dyshomeostasis on lipid metabolism and atherosclerotic progression. Excess copper triggers the Fenton reaction, which induces mitochondrial lipid metabolism disorders. It also modulates PDHA1 and PI3K-Akt-mTOR signaling to promote lipid metabolism disorders and PDE3B (promoting cAMP-mediated lipolysis). Excessive lipids further disrupt copper transporters/chaperones, forming a vicious cycle. Accumulated lipids drive OX-LDL production, ultimately leading to atherosclerosis. Conversely, deficient copper impairs SREBP 1/2 activity, reducing triglyceride hydrolysis and fatty acid β-oxidation (also contributing to lipid dysregulation).

Copper Overload promotes cardiomyocyte injury by inducing oxidative stress, which in turn disrupts lipid metabolism 36. The hydroxyl radicals produced via copper-catalyzed Fenton reactions mediate oxidative damage, primarily by oxidizing LDL into oxidized LDL (ox-LDL), which serves as a key driver of atherosclerotic plaque formation 121. Moreover, copper influences lipid signaling by modulating the activity of phosphodiesterase PDE3B, an enzyme that hydrolyzes cyclic adenosine monophosphate (cAMP) to regulate lipolysis 122. The cuproptosis-related gene PDHA1 may serve as a node linking these pathways, regulating lipid metabolism through its interaction with the PI3K-Akt-mTOR signaling pathway 123 **(Figure 5)**.

The lipid composition and fluidity of cell membranes affect the efficiency of the primary copper importer SLC31A1 (CTR1), thereby influencing the intracellular copper balance 124. Excessive lipid accumulation disrupts copper homeostasis by altering the expression and localization of key copper transporters and chaperones 125. The lipid microenvironment can also modulate susceptibility to cuproptosis. Under certain conditions, it may synergize with copper chaperones to facilitate efficient copper transport, thereby preventing cuproptosis 126. More broadly, alterations in lipid metabolism, such as those mediated by short-chain fatty acids (e.g., butyric acid), can influence cell death pathways by modulating the expression of copper-related genes 127. This establishes a feedback loop, as copper is an essential cofactor for numerous lipid metabolic enzymes; thus, initial copper deficiency can directly disrupt lipid homeostasis, which, in turn, further perturbs copper handling 116.

### Central Role of Mitochondrial Metabolism in Cardiovascular Disease and Cardiac Cuproptosis

Mitochondrial metabolism serves a dual role in the cardiovascular system: it is the primary energy source for cardiomyocytes and a central integrator of overall cardiac health, and its dysfunction is a key driver of disease pathogenesis 128. This centrality is underscored by the heart's exceptional dependence on mitochondrial oxidative phosphorylation, which supplies approximately 95% of the ATP required for contraction 23. Consequently, mitochondrial dysfunction has been identified as a central contributor to the onset and progression of various cardiovascular diseases, including HF, myocardial ischemia/reperfusion (I/R) injury, and atherosclerosis. The progressive decline in mitochondrial function, characterized by impaired ATP synthesis, excessive ROS production, and structural abnormalities, triggers a vicious cycle involving energy crises, oxidative stress, and inflammatory responses, ultimately accelerating cardiomyocyte death 129, 130.

At the molecular level, this dysfunction mediates pathology via several interconnected pathways. The mitochondrial electron transport chain (particularly Complexes I and III) is a major site of ROS generation. Beyond directly damaging mitochondrial components, ROS oxidize LDL to form ox-LDL, which promotes atherosclerosis by driving macrophage-to-foam cell formation 131, 132. ROS can activate pro-inflammatory pathways, such as NF-κB, thereby inducing cardiomyocyte hypertrophy, apoptosis, and myocardial fibrosis, which further exacerbate cardiac structural and functional impairments 133.

Mitochondrial calcium (Ca²⁺) homeostasis disorder represents another crucial mechanism that exhibits close crosstalk with the ROS pathway. As a key regulator of ATP synthesis, Ca²⁺ modulates enzymes such as pyruvate dehydrogenase. Abnormal Ca²⁺ influx impairs energy production and activates caspase proteins, initiating the intrinsic apoptotic pathway and accelerating cardiomyocyte loss 134, 135. Notably, excessive ROS can further exacerbate calcium homeostasis disorders by damaging the sarcoplasmic reticulum Ca²⁺-ATPase and mitochondrial calcium uniporter, forming a malignant cascade of “excessive ROS-calcium imbalance-energy crisis” 136.

Of particular relevance, the heart is exquisitely vulnerable to copper imbalance owing to its profound reliance on mitochondrial energy production—organelles central to both copper homeostasis and the execution of cuproptosis 75, 137. The initiation of cuproptosis critically depends on mitochondrial oxidative stress, as the oxidation of lipoyl groups on target proteins is a prerequisite for their high-affinity binding to Cu⁺, which drives toxic protein aggregation 3. The accumulated Cu⁺ catalyzes intramitochondrial Fenton-like reactions, generating a burst of mtROS and amplifying oxidative stress. This creates feed-forward cycles that exacerbate cuproptosis: mtROS oxidize cytosolic GSH, releasing bound Cu⁺, and may upregulate the copper importer CTR1 via pathways such as NF-κB, increasing copper influx 65. The resulting oxidative stress promotes Drp1-mediated mitochondrial fission, mPTP opening, and membrane depolarization, thereby amplifying apoptotic signaling 52, 138. The copper-induced ROS burst, coupled with disrupted TCA cycle-derived GSH precursors, leads to irreversible GSH depletion 108, 139. This inactivates GPX4, halting lipid peroxide clearance and driving cell death 140.

**Figure 6:**
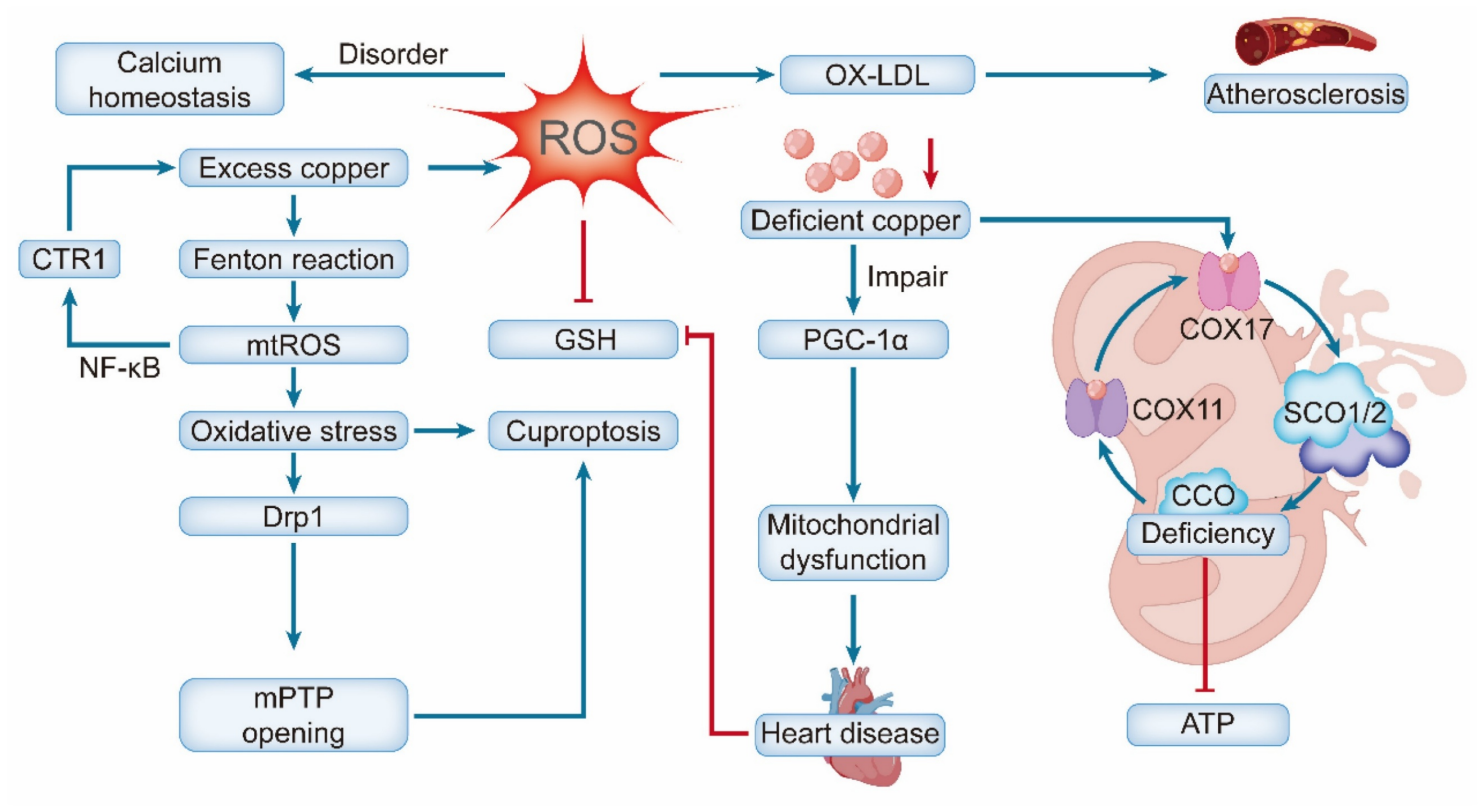
Copper dyshomeostasis in heart disease via mitochondrial pathways. Excess copper enters via CTR1, and excess ROS reduces GSH and drives OX-LDL production, contributing to atherosclerosis, Drp1-mediated fission, mPTP opening, and ultimately cuproptosis. Copper deficiency impairs mitochondrial biogenesis (PGC-1α) and disrupts COX assembly (via COX17, SCO1, and SCO2), resulting in bioenergetic failure due to ATP deficiency.

Conversely, copper deficiency impairs cardiac mitochondria via distinct mechanisms. It disrupts the copper chaperone system (e.g., COX17, SCO1/SCO2), drastically reducing CCO biosynthesis and activity. This compromises ATP production and myocardial oxygen consumption rate, which is vital for contractile function 46, 141. Deficiency also impairs PGC-1α, the master regulator of mitochondrial biogenesis. Notably, both its loss and uncontrolled overexpression are destructive, highlighting the need for precise regulation to maintain cardiac metabolism 142, 143. Biochemically, these failures manifest as enlarged and degenerated mitochondria with loss of cristae, driving pathological cardiac remodeling 144. A self-amplifying vicious cycle lies at the heart of this pathology. Pre-existing mitochondrial dysfunction due to aging, diabetes, or ischemia creates a permissive environment for cuproptosis by depleting GSH and reducing SOD2 activity, thereby compromising the organelle's ability to chelate copper and neutralize mtROS 145, 146. This expands the labile Cu⁺ pool and lowers the threshold for cuproptosis initiation. In turn, cuproptosis inflicts further mitochondrial damage, disrupting membrane integrity and metabolic pathways 147. This reciprocal relationship establishes a bidirectional causal link that accelerates the pathogenesis of cardiovascular diseases** (Figure 6)**.

To counteract this vicious cycle, the heart is dependent on a robust mitochondrial quality-control system. This system replenishes healthy mitochondria through biogenesis, maintains network integrity via dynamics (fusion/fission), and clears damaged units through mitophagy 148. Given their pivotal roles, targeting mitochondrial metabolism and copper homeostasis presents a promising strategy for novel therapeutic interventions in several cardiovascular diseases.

### Cell-type-specific Cuproptosis in Cardiovascular System

The cardiovascular system comprises diverse cell types—including cardiomyocytes, ECs, VSMCs, and fibroblasts—each exhibiting distinct susceptibilities and responses to cuproptosis 149. This heterogeneity is primarily determined by three interlinked factors: mitochondrial abundance, metabolic activity, and intrinsic copper handling capabilities. Consequently, cell types with high mitochondrial respiration, the primary target of copper toxicity, are disproportionately vulnerable 80.

Cardiomyocytes are the primary site of copper-induced injury because of their immense reliance on mitochondrial metabolism. The high density of mitochondria provides numerous targets for copper binding to lipoylated TCA cycle enzymes, directly disrupting the energy supply vital for contraction 75. This intrinsic vulnerability is compounded by a weak copper export system, characterized by low expression of ATP7A/B transporters, which facilitates toxic accumulation 100. In pathologies such as myocardial I/R injury, this confluence of factors leads to extensive cuproptosis, directly worsening systolic function 150.

ECs exhibit significant susceptibility, which is dictated more by their physiological context than their metabolic rate. Their direct contact with circulating copper and pro-oxidants (e.g., ox-LDL), combined with limited copper export capacity, creates a precarious copper balance 151, 152. Given their role as central regulators of vascular homeostasis, EC cuproptosis can initiate widespread dysfunction, impairing barrier integrity and vasoreactivity 153.

The susceptibility of VSMCs is not fixed but is a direct function of their phenotypic state. Differentiated contractile VSMCs are relatively resistant. In contrast, synthetic, proliferative VSMCs—which dominate pathologies such as atherosclerosis—undergo a metabolic shift toward glycolysis and possess reduced antioxidant defenses, rendering them highly vulnerable to cuproptosis 154, 155. This phenotypic targeting makes cuproptosis a potential modulator of vascular remodeling.

Fibroblasts are the most resistant cell type, protected by their low mitochondrial content and high expression of copper-buffering proteins, such as MTs 12. However, this resilience does not render them inert. Instead, they are activated by damage-associated molecular patterns released from neighboring cardiomyocytes or ECs undergoing cuproptosis. Thus, paradoxically, fibroblast resistance fuels chronic disease progression by promoting pathological fibrosis 156.

The cell-type-specific impact of cuproptosis creates a dualistic injury pattern: it directly causes acute loss of contractile and endothelial function by eliminating cardiomyocytes and ECs, while simultaneously driving chronic maladaptive remodeling through the activation of resistant VSMCs and fibroblasts 154, 157. This refined understanding underscores the necessity of cell-type-targeted therapeutic strategies that either protect vulnerable cells or modulate the response of resistant cells in cardiovascular diseases.

## Role of Copper Homeostasis and Cuproptosis in Cardiovascular Disease

**Figure 7:**
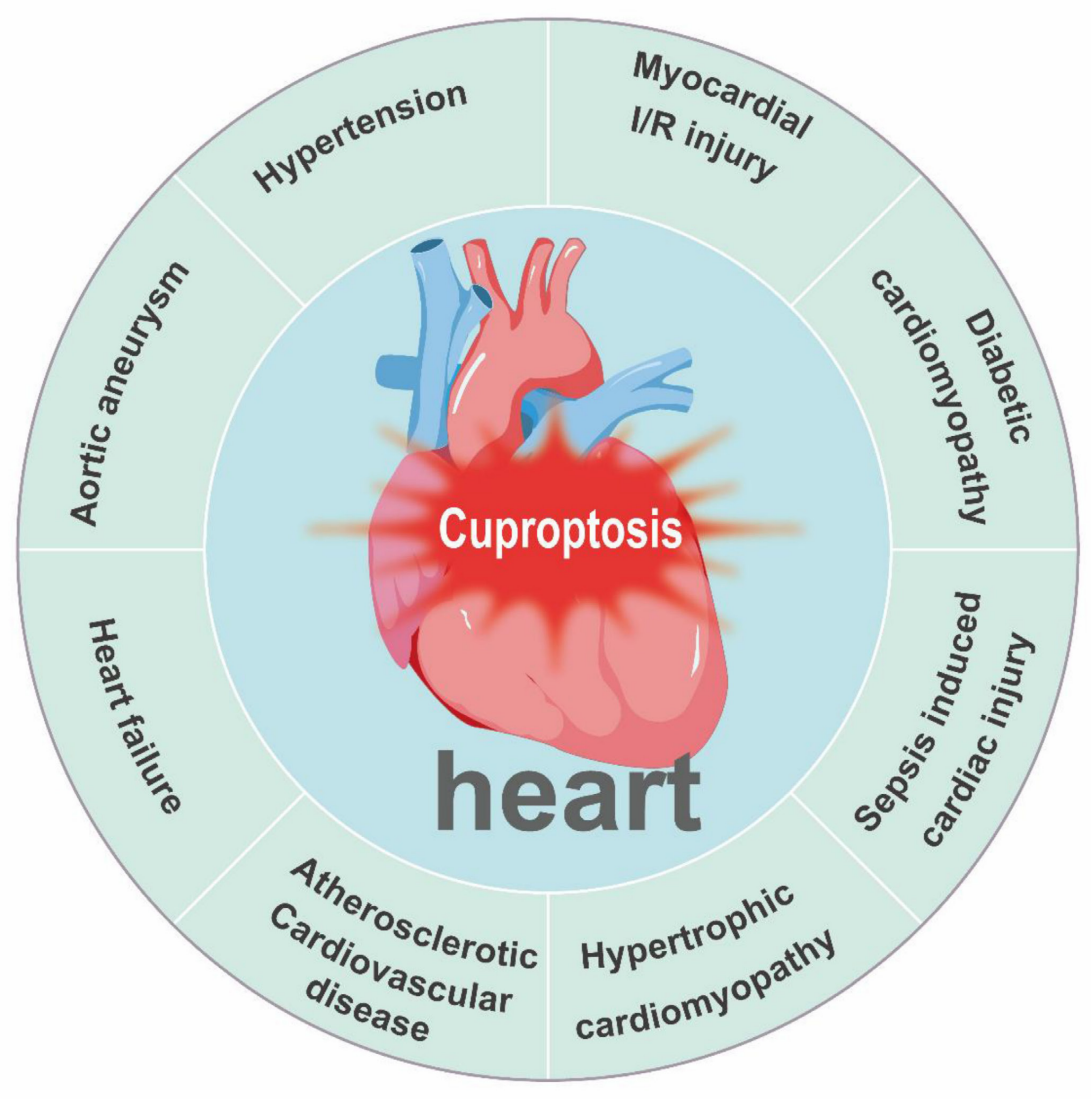
Cuproptosis is associated with major cardiac and vascular diseases, including myocardial ischemia/reperfusion injury, diabetic cardiomyopathy, sepsis-induced cardiac injury, hypertrophic cardiomyopathy, atherosclerotic cardiovascular disease, heart failure, aortic aneurysm, and hypertension.

Copper acts as a quintessential “double-edged sword” in cardiovascular biology. Although it serves as an essential cofactor for critical enzymes governing antioxidant defense, energy production, and structural integrity, its dysregulation—manifesting as either deficiency or excess—is a common pathogenic thread across diverse cardiovascular conditions. These include myocardial I/R injury, diabetic cardiomyopathy (DCM), sepsis-induced cardiac injury, hypertrophic cardiomyopathy, atherosclerotic cardiovascular disease, HF, aortic aneurysm (AA), and hypertension **(Figure 7)**. In each of these pathologies, disrupted copper homeostasis contributes to disease progression through distinct but convergent molecular mechanisms **(Table 2)**.

### Copper Homeostasis and Cuproptosis in Myocardial ischemia/reperfusion Injury

Copper homeostasis is critically involved in the pathophysiology of myocardial I/R injury, with both deficiency and excess exacerbating damage through distinct mechanisms 158. The acute inflammatory and oxidative stress that characterizes I/R injury is profoundly influenced by cellular copper status 159.

Copper deficiency impairs the heart's intrinsic defense mechanisms, thereby exacerbating I/R injury. It disrupts antioxidant defenses by reducing the activity of copper-dependent enzymes, such as CCS, leading to uncontrolled oxidative stress and intensified inflammatory responses 160, 161. Chronic deficiency may elevate the baseline risk of ischemia by impairing vascular elasticity (via compromised LOX function) and promoting platelet aggregation 162. Mechanistically, aberrant copper homeostasis during I/R impairs Fe-S cluster integrity, increases ROS production, and depletes GSH, culminating in synergistic proteotoxic and oxidative damage 160. Supporting this, therapeutic strategies aimed at correcting deficiencies or restoring copper-dependent signaling have shown promise. In preclinical models of I/R injury, copper supplementation improves functional recovery by mitigating oxidative damage and enhancing mitochondrial bioenergetics 96. In animal models, low-dose copper sulfate intake exerts potent antioxidant, anti-inflammatory, and anti-proliferative effects, attenuating I/R-induced tissue damage 163. Similarly, controlled administration of copper ions can protect against tissue death by mitigating oxidative stress and inflammation, as evidenced by reduced lipid peroxidation and enhanced antioxidant reserve levels 164.

In contrast, copper overload causes damage through direct cytotoxic mechanisms. Excess copper can promote ferritin depletion and disrupt iron metabolism, thereby sensitizing cardiomyocytes to ferroptosis, a process that synergistically aggravates I/R injury 67. This is supported by clinical observations; serum copper and CP levels rise post-myocardial infarction, indicating a stress response that, when excessive, may transition from a compensatory mechanism to a contributor of damage 165. In summary, maintaining copper levels within a strict physiological range is paramount for mitigating I/R injury. Deficiency compromises intrinsic defensive capacity, whereas overload directly instigates oxidative and proteotoxic stress, including crosstalk with ferroptosis 166. Therapeutic interventions must be precisely calibrated to restore homeostasis without inducing toxicity.

### Copper Homeostasis and Cuproptosis in Diabetic cardiomyopathy

DCM is characterized by a pathological triad of cardiomyocyte death, interstitial fibrosis, and structural remodeling, culminating in HF 167. Beyond the established mechanisms, a pivotal contributor to DCM pathogenesis is the disruption of systemic and cellular copper homeostasis, which primarily inflicts damage through the aberrant compartmentalization of copper within cardiac tissues 80.

The imbalance in copper metabolism in DCM presents a paradox. Systemic diabetes is associated with elevated plasma and urinary copper concentrations; however, the myocardium exhibits a marked reduction in copper content 168. This discrepancy arises from defective myocardial copper handling, as evidenced by impaired expression and trafficking of key copper chaperones, which disrupts mitochondrial copper delivery in diabetic hearts 169. The consequent sequestration of redox-active Cu²⁺ in the extracellular compartment and certain intracellular pools is a key mediator of cytotoxicity and cardiotoxicity in diabetes 51, 170. Evidence from human studies and experimental models has linked cuproptosis to DCM. Mechanistically, high-glucose-induced AGEs promote cuproptosis in cardiomyocytes by upregulating SLC31A1 expression, which drives pathogenic Cu⁺ accumulation 80. This extracellular and mislocalized intracellular copper exacerbates DCM through multiple pathways, activating the TGF-β/Smad signaling pathway and amplifying oxidative stress, collectively promoting extracellular matrix deposition and fibrotic remodeling 171.

Given that direct coronary perfusion of low-concentration copper impairs cardiac function, dysregulation of copper homeostasis presents a clear and actionable therapeutic target. Accordingly, the copper chelator triethylenetetramine (TETA) has shown efficacy in markedly improving cardiac performance in preclinical models of DCM 80. These findings underscore the fact that the core pathology stems from dysregulated copper transport and tissue distribution, not dietary copper intake 169. Consequently, the therapeutic promise for DCM lies not in generic copper restriction but in strategies capable of rectifying this specific copper mislocalization. Approaches such as targeted chelation or restoration of chaperone function present a rational and promising avenue for halting DCM progression.

### Copper Homeostasis and Cuproptosis in Sepsis-induced Cardiac Injury

Sepsis-induced cardiac dysfunction is a primary cause of death and a major determinant of poor long-term outcomes 172. Its pathogenesis involves excessive oxidative stress, dysregulated inflammatory cascades and profound mitochondrial damage 173. Beyond these established factors, emerging evidence suggests that the disruption of copper homeostasis and the induction of cuproptosis are critical integrative mechanisms that potentially link systemic inflammatory-metabolic stress to direct cardiomyocyte death 174.

The relevance of this pathway is underscored by the significant dysregulation of cuproptosis-related genes in experimental models of septic cardiomyopathy 126. The septic milieu itself—characterized by a cytokine storm and metabolic acidosis—acts as a potent upstream disruptor of copper homeostasis, priming the heart for copper-dependent injury 175. This dysregulation is systemically reflected by a marked elevation in CP, a key copper-transporting acute-phase protein and a recognized clinical biomarker of sepsis 176. However, the pathophysiological role of elevated CP levels may be dualistic. Although it has protective antioxidant functions, its surge may also alter copper kinetics, potentially increasing the delivery of redox-active copper to tissues or disrupting its cellular utilization, thereby paradoxically exacerbating copper-mediated toxicity in cardiomyocytes 177. The elucidation of cuproptosis as a mechanism connecting inflammatory, metabolic, and mitochondrial damage to cardiomyocyte death opens new therapeutic avenues. Consequently, targeting this pathway—for instance, by modulating systemic copper distribution or using specific cuproptosis inhibitors—represents a promising strategy for cardioprotection in sepsis.

### Copper Homeostasis and Cuproptosis in Hypertrophic Cardiomyopathy

Copper deficiency is a well-established causative factor of cardiac hypertrophy, which manifests as concentric thickening of the ventricular walls and interventricular septum, resembling pressure-overload hypertrophy 178. This pathology is primarily driven by severe mitochondrial dysfunction, which impairs energy production and triggers compensatory hypertrophic responses 179. This process is exacerbated by dysregulated vascular endothelial growth factor (VEGF) and ROS generated via the Fenton reaction, collectively promoting maladaptive remodeling 180.

In a pressure overload-induced cardiomyocyte hypertrophy model, COX17 deletion inactivated CCO. This mitochondrial respiratory defect likely secondarily impairs MFN1-mediated mitochondrial fusion, potentially through mechanisms involving energy depletion or elevated oxidative stress 52. Similarly, mutations in the SCO2 gene impair mitochondrial function and can cause cardiomyopathy, underscoring the critical role of copper delivery systems 181. Beyond bioenergetics, copper ions are essential cofactors for specific signaling pathways. For instance, copper is required for the activation of MEK1 kinase, and disruption of this copper-dependent signaling contributes directly to myofibrillar disorganization and pathological hypertrophy 182, 183.

In the context of dietary copper deficiency, supplementation can restore cardiac copper levels. This restoration leads to improved mitochondrial health, enhanced CCO activity, angiogenesis (via VEGF signaling), and attenuation of cardiac hypertrophy 35, 178. In cases of specific chaperone deficiencies (e.g., SCO2 mutations), copper-histidine therapy can improve cardiac function by restoring copper availability 184. Targeting the downstream effectors of copper-dependent pathways is another strategy. For instance, MEK1 inhibitors have been shown to improve cardiac function and reverse myocardial fibrosis and hypertrophy 185. In pathological states where localized or systemic copper excess contributes to hypertrophy, copper chelators can alleviate oxidative stress and subsequent remodeling 12.

### Copper Homeostasis and Cuproptosis in Atherosclerotic Cardiovascular Disease

Atherosclerosis, a chronic inflammatory disease of the arterial wall, is characterized by the accumulation of lipids, inflammatory cells, and extracellular matrix, leading to plaque formation 186. Copper homeostasis plays a complex, context-dependent role in this pathology, with both excess and deficiency contributing to disease progression via distinct mechanisms 187, 188.

Intracellular copper accumulation is a key feature of atherosclerosis. In atherosclerotic cells, such as ox-LDL-treated macrophages, copper levels are significantly elevated (by approximately 62%), providing a catalyst for redox reactions 154. Through the Fenton reaction, redox-cycling copper ions potently catalyze hydroxyl radical formation, causing DNA damage and propagating lipid peroxidation 3. Serum Cu²⁺ also drives the oxidation of LDL to form copper-ox-LDL, a key ligand that binds to the LOX-1 receptor on ECs to initiate plaque formation 189. Furthermore, copper stimulates acute-phase proteins and activates the pro-inflammatory transcription factor NF-κB via ROS generation. This cascade promotes vascular inflammation, a process further amplified by upregulated CP expression 190.

Paradoxically, copper-dependent processes can also impair VSMC accumulation within the neointima, thereby affecting plaque stability 191, 192. Moreover, copper homeostasis critically regulates the migration of VSMCs. The release of free copper ions, facilitated by transporters such as ATP7A and ATOX1, induces neointimal thickening following vascular injury, contributing directly to lesion development 193, 194. In human studies, the expression level of the copper transporter SLC31A1 was directly correlated with atherosclerotic plaque vulnerability 42.

The pathogenic role of copper is context-dependent and exhibits a U-shaped risk curve. As described above, excess copper drives oxidative and inflammatory injuries. Conversely, copper deficiency elevates the risk of atherosclerosis by increasing cholesterol levels and reducing the activity of the copper-dependent antioxidant enzyme SOD1. This diminishes the overall antioxidant defenses and reduces NO bioavailability, leading to endothelial dysfunction 187. The intertwined dysregulation of copper and iron metabolism further exacerbates the disease by promoting apoptosis in lipid-rich foam cells and VSMCs, which are particularly vulnerable to metal-dependent cell death 195, 196.

Epidemiological evidence shows that a high dietary copper intake is associated with a reduced incidence of coronary heart disease 39. In animal models of copper deficiency, copper supplementation mitigated atherosclerotic lesion progression, as evidenced by reductions in lesion area, endothelial cell death, and plasma cholesterol levels 188. Conversely, given the pro-oxidant and pro-inflammatory effects of excess copper within plaques, preclinical studies have reported that strategies utilizing copper chelators may hold potential for attenuating plaque progression, although this requires further investigation. Additionally, copper-related DNA methylation alterations are linked to an elevated risk of acute coronary syndrome, suggesting an epigenetic role in the disease 197, 198.

### Copper Homeostasis and Cuproptosis in Heart Failure

HF, a complex clinical syndrome characterized by significant functional impairment and high morbidity, stems from impaired cardiac contractility and/or ventricular filling 199. A growing body of evidence underscores the critical role of disrupted copper homeostasis in its pathogenesis, wherein both excess and deficiency of copper significantly contribute to disease progression 30.

A meta-analysis conducted by Liu* et al.* 200 confirmed that elevated serum copper levels have been repeatedly are associated with an increased risk of HF. A meta-analysis of 1,504 individuals confirmed a positive association between high serum copper levels and HF 201. Clinically, elevated copper levels and their carrier protein CP are significant predictors of adverse outcomes, including mortality, in patients with HF 202. The underlying pathophysiology involves intracellular copper accumulation in cardiomyocytes, which directly induces cuproptosis 100. Copper overload impairs mitochondrial electron transport, leading to excessive ROS production and a diminished antioxidant capacity. This is notably exacerbated by increased hydrogen peroxide production at the flavin site of mitochondrial complex IV 203. The resultant oxidative damage triggers an inflammatory response characterized by the upregulation of acute-phase proteins, such as CP, further exacerbating cardiac dysfunction 204.

Paradoxically, copper deficiency worsens HF. Experimental studies have demonstrated that a copper-deficient diet exacerbates myocardial dysfunction in HF models 205. In mouse models, copper deficiency impairs cardiac function, manifesting as reduced diastolic and systolic performance alongside a diminished inotropic response to beta-adrenergic stimulation, which compromises the heart's ability to adapt to stress 206.

This dual pathophysiology suggests the need for targeted therapeutic strategies. In states of copper overload, interventions that remove excess copper or restore normal cellular copper transport have shown promise in improving cardiac and mitochondrial function in preclinical studies 51. In contrast, copper supplementation can fully restore cardiac function and normalize the β-adrenergic response in deficiency states 206. In summary, maintaining precise copper homeostasis is essential for cardiac function. Therapeutic strategies that correct specific imbalances—whether through chelation in overload or supplementation in deficiency—hold significant promise for mitigating HF progression.

### Copper Homeostasis and Cuproptosis in Aortic Aneurysm

AA, a life-threatening pathological dilation of the aortic wall and the ninth leading cause of death globally, arises from a complex interplay of factors, including inflammation, apoptosis, and oxidative stress 207. Emerging evidence firmly establishes that the disruption of copper homeostasis is a significant contributor to this process, driving aortic wall degeneration through interconnected pathways involving oxidative damage, inflammation, vascular dysfunction, and metabolic defects 208.

A pivotal initiating event is the downregulation or dysfunction of the key copper exporter, ATP7A. This defect leads to intracellular copper accumulation, exacerbating local oxidative stress and promoting aneurysm progression. Part of this effect is mediated by the upregulation of miR-125b, which amplifies copper-dependent pro-inflammatory signaling 209. The accumulated copper ions function as potent catalysts for destructive processes within the aortic wall. They directly drive lipid peroxidation and inhibit essential enzymes, compromising vascular integrity 210. Furthermore, copper imbalance disrupts the critical equilibrium that governs NO synthesis and degradation. As NO is a central regulator of vascular tone and remodeling, this disruption promotes the pathological dilation characteristic of aneurysms 211. Underlying mitochondrial defects, often linked to disturbances in the TCA cycle, can be worsened by copper imbalance, further contributing to the energetic deficit and cellular stress that propel AA progression 212. The delineation of these copper-mediated pathways has revealed promising therapeutic targets. Strategies aimed at correcting copper transport (e.g., by restoring ATP7A function) or mitigating the downstream consequences of excess copper (e.g., using targeted antioxidants) could potentially slow or halt aortic wall degeneration.

### Copper Homeostasis and Cuproptosis in Hypertension

Clinical and experimental evidence confirms a U-shaped relationship between copper levels and blood pressure, indicating that both copper deficiency and excess are associated with a higher prevalence of hypertension 213. This duality reflects the distinct pathogenic mechanisms at each extreme of copper homeostasis.

On the one hand, copper deficiency promotes hypertension primarily through impaired antioxidant defense and vascular dysfunction. Deficiency inactivates the copper-dependent antioxidant enzyme SOD1, leading to increased vascular oxidative stress. This oxidative environment uncouples endothelial NO synthase, reducing NO bioavailability and impairing vasodilation—a direct pathway to increased vascular resistance 214, 215. Furthermore, as copper is a physiological inhibitor of angiotensin-converting enzyme, its deficiency disinhibits the renin-angiotensin-aldosterone system, further exacerbating hypertension 216, 217. Consistent with this mechanistic link, lower blood copper levels have been observed in hypertensive animal models 215.

Copper dysregulation and excess copper also contribute to disease progression. In the vasculature, cuproptosis directly induces EC death and drives VSMCs toward a pro-inflammatory synthetic phenotype. This dual insult exacerbates endothelial dysfunction and promotes pathological vascular remodeling and sclerosis, creating a vicious cycle that elevates blood pressure 198, 218. In the heart under pressure overload, intracellular copper accumulation can trigger cardiomyocyte cuproptosis via FDX1 activation, leading to lipoylated protein aggregation and mitochondrial damage, thereby exacerbating cardiac injury 12.

Population-based studies have corroborated this complex relationship. A cross-sectional study reported demographic variations and a potential independent association between serum copper and hypertension 219. Notably, the protective negative association between dietary copper intake and myocardial infarction appears particularly strong among patients with hypertension, underscoring the clinical importance of maintaining optimal copper status in this high-risk group 39. In summary, precise copper homeostasis is crucial for maintaining normal vascular tone and cardiac health, and deviations in either direction contribute to the pathogenesis and complications of hypertension.

## Clinical Application of Copper Targeting Strategy for Cardiovascular Disease

Targeting dysregulated copper homeostasis represents a promising frontier in cardiovascular therapy, offering a mechanistic approach that is distinct from conventional treatments. Current strategies aim to restore balance through two principal means: chelation to mitigate copper overload or supplementation to correct deficiency, both with the goal of re-establishing metabolic equilibrium. In contrast, conventional therapies, such as β-blockers, statins, and ACEIs/ARBs, primarily target neurohormonal pathways, lipid metabolism, and hemodynamics 220 **(Table 3)**. Therefore, modulating copper homeostasis and cuproptosis addresses a novel pathogenic axis that is not directly targeted by existing first-line drugs, suggesting the potential for synergistic or personalized therapeutic strategies.

### Copper Chelators

Copper chelators are therapeutic agents that bind copper ions to form stable complexes, alleviating cardiovascular pathologies, primarily by reducing the labile intracellular copper pool. This inhibits cuproptosis and associated oxidative damage by preventing ROS generation 80 **(Table 4)**. Furthermore, the reduction in bioavailable copper impairs the function of copper-dependent proteins and signaling pathways, including SOD1, copper chaperones (ATOX1, ATP7A), and transcription factors, such as HIF-1α and NF-κB 221. The overarching cardioprotective effect is achieved through the restoration of mitochondrial integrity and function, as evidenced by the recovery of key components (e.g., SCO1, CCO, and SOD1) and upregulating the biogenesis regulator PGC-1α 51, 204. The benefits of this approach have been validated in several disease models. Copper chelation inhibits pivotal VSMC migration in intimal hyperplasia 194 improves recovery after myocardial infarction 222 and reduces neointimal formation and atherosclerosis in ApoE-deficient mice 223. Overall, by chelating excess copper, these agents help to restore metabolic homeostasis and protect cardiovascular cells.

Tetrathiomolybdate (TTM) is a highly selective copper chelator that prevents copper accumulation and toxicity *in vivo* by sequestering excess copper ions 224. TTM inhibits their uptake and delivery by chaperone proteins to downstream cuproenzymes, such as LOX, inducing functional defects in these enzymes 225. The efficacy of TTM stems from its ability to modulate copper bioavailability across diverse cardiovascular disease models. It attenuates atherosclerosis in ApoE⁻/⁻ mice by reducing bioavailable copper and suppressing vascular inflammation 198 and protects against AAs in ATP7A-deficient mice by inhibiting endothelial ROS 209. In the heart, TTM improves myocardial injury in diabetes and after ischemia by enhancing mitochondrial protein activity and reducing infarct size 80. It also alleviates chronic stress-induced myocardial fibrosis by regulating the cardiac copper levels 49. Additionally, TTM inhibits TNF-α-induced activation of NF-κB and AP-1, leading to the suppressed expression of adhesion molecules (VCAM-1 and ICAM-1) and the chemokine MCP-1. This attenuates endothelial activation and contributes to the inhibition of atherosclerotic progression 226, 227.

TETA, typically administered as a dihydrochloride salt, restores cardiac function by rectifying copper homeostasis and repairing mitochondrial integrity. Its primary mechanism involves restoring the activity of key mitochondrial enzymes, such as SOD1, CCS, and CCO, thereby re-establishing metabolic function 51. Animal studies have validated this efficacy, showing that TETA therapy enhances cardiac pumping efficiency and normalizes the levels of copper, copper-binding proteins, and CCO in the myocardial tissue 51. Furthermore, it ameliorates hypertrophic cardiomyopathy and reverses diabetes-induced mitochondrial damage by restoring the expression and function of crucial cardiac energy metabolic proteins 37, 228.

### Small-molecule Inhibitors of Copper Chaperone Proteins

The clinical utility of conventional copper chelators is limited by dose-dependent toxicities, primarily stemming from systemic copper deficiency and off-target chelation of essential metals, such as zinc and iron. This underscores the pressing need for novel agents capable of selectively modulating intracellular copper homeostasis without systemic depletion 46. Emerging strategies focus on precise molecular targets within copper transport machinery.

A key approach involves the development of small molecules that directly inhibit intracellular copper trafficking. The drug DCAC50 exemplifies this strategy by binding to the copper chaperones ATOX1 and CCS, thereby disrupting copper delivery and its dependent signaling without harming normal cells 229, 230. The copper chaperone ATOX1 is a particularly compelling target in this context. Beyond its cytoplasmic transport role, ATOX1 is critically involved in vascular pathology; it promotes VSMC migration—a key process in neointimal and atherosclerotic lesion formation—and recruits inflammatory cells 93, 194. Mechanistic insights have revealed that ATOX1 translocates to the nucleus in response to inflammatory cytokines or copper ions 93. Moreover, in TNF-α-stimulated ECs, the copper-dependent ATOX1-TRAF4 interaction promotes nuclear translocation and initiates ROS-dependent inflammatory responses, solidifying this axis as a promising therapeutic target 45.

In contrast, a distinct pharmacological strategy employs copper ionophores, such as elesclomol. Instead of inhibiting copper flux, these agents form complexes with copper to facilitate cellular uptake. Their pro-oxidative action exploits copper to induce selective toxicity in target cells, representing a different therapeutic logic that is useful in specific contexts 231, 232. Together, these approaches—precise inhibition of copper chaperones and conditional use of copper ionophores—represent innovative strategies for targeting copper dyshomeostasis in cardiovascular and other diseases.

### Copper Ionophore

Copper ionophores are small molecules that enhance intracellular copper bioavailability by shuttling copper across the cell membrane. Primarily used to treat research overload or exploit copper toxicity in therapy, this class includes disulfiram and pyrithione 233.

Their intracellular effects are complex and context dependent. Some ionophores can increase NO levels and reduce pro-inflammatory cytokine levels, indicating their anti-inflammatory potential 234. Others, such as members of the 8-hydroxyquinoline family, can synergize with SOD transporters to mitigate intracellular ROS, thereby protecting against oxidative stress and apoptosis 235. A central therapeutic challenge is their lack of cell-type specificity, which leads to off-target effects 236. To overcome this limitation, targeted delivery systems are being developed. For example, Su *et al.* 237 conjugated N-acetylgalactosamine to a copper ionophore to create Gal-Cu, a liver-targeting system. This design enhances copper delivery to hepatocytes while reducing exposure to nontarget organs, thereby minimizing systemic toxicity.

However, the use of ionophores remains a pharmacological double-edged sword. Cells possess adaptive homeostatic mechanisms, such as upregulation of the copper efflux transporter ATP7B, to counteract increased influx 238. Excessive ionophore administration can overwhelm these defenses, leading to systemic copper poisoning, characterized by hepatotoxicity, nephrotoxicity, and hematopoietic suppression 239. Therefore, the therapeutic window for ionophores is narrow, and precision in their delivery and dosing is paramount.

## Limits and Challenges

Conventional copper-modulating therapies, including chelators (e.g., TTM and TETA) and the ionophore elesclomol, are fundamentally limited by their lack of specificity, which constrains their clinical utility and leads to significant adverse effects. The most critical drawback is the inability to precisely target diseased tissues or cells. Chelators non-selectively deplete systemic copper, harming healthy organs that require copper for normal function and leading to systemic deficiency 240. Conversely, ionophores such as elesclomol lack cellular specificity, delivering copper indiscriminately and risking overload in healthy tissues 241. Furthermore, many chelators lack selectivity for copper over other essential metals, causing off-target depletion of ions such as zinc and iron, which compounds their toxicity 242.

The non-selective action of these agents impairs the functions of vital cuproenzymes. In cardiomyocytes, a reduction in CCO activity compromises mitochondrial energy production 243. Systemically, copper deficiency disrupts iron metabolism by impairing CP ferroxidase activity 244. The most common clinical manifestations of this broad physiological disruption are bone marrow suppression and anemia. Anemia is multifactorial, arising from defective CCO activity in erythrocyte precursors (normocytic anemia) and, in the case of TTM, from CP dysfunction, leading to iron retention and sideroblastic features 224, 244. Some chelators, such as D-penicillamine, also cause direct neurotoxicity, likely by crossing the blood-brain barrier and disrupting copper-dependent central nervous system enzymes, impairing energy metabolism, and increasing oxidative stress 245, 246.

These therapies have a narrow therapeutic window, indicating that their efficacy and toxicity are critically dose dependent. For example, excessive doses of the copper ionophore elesclomol can flood cells with copper, overwhelming antioxidant defenses and triggering lethal oxidative stress 247 whereas excessive copper supplementation can cause overload and ROS-mediated damage 248. Moreover, patient-specific factors significantly alter the risk. In DCM, upregulation of the copper importer SLC31A1 makes patients exceptionally sensitive to chelators, whereby a standard dose can precipitate severe deficiency 240. Concurrent liver dysfunction, by impairing copper excretion, further increases the risk of accumulation and adverse cardiovascular events 242, 249.

Conventional *in vitro* systems, such as primary cardiomyocytes and H9c2 cell lines, have significant limitations in studying copper metabolism. They lack the systemic context necessary to recapitulate inter-organ crosstalk (e.g., hepatic regulation of cardiac copper) and are inherently static, making them unsuitable for modelling the gradual pathogenesis of chronic copper imbalances. Consequently, their utility is largely confined to studies on acute toxicity or transient metabolic alterations 250, 251. The simplified 2D microenvironment—devoid of a 3D matrix, vasculature, and key factors such as CP—further leads to dysregulated copper transporter expression and non-physiological toxicity responses 252. Although animal models provide a more integrated physiological context, their translational value is limited by interspecies differences in copper metabolism and the difficulty in modelling human-specific gene-environment interactions 253. A major shortcoming is their frequent failure to replicate human comorbidities (e.g., hypertension and diabetes), which profoundly alters copper homeostasis and cell death pathways. This discrepancy likely underlies the repeated failure of copper-targeted interventions in clinical translation 254. Thus, a critical appreciation of these inherent limitations at each stage—from cell culture to animal models—is paramount for rigorously interpreting data and designing studies with genuine clinical relevance.

## Future Directions

Building upon a critical understanding of the limitations inherent in current therapies and research models, the future of this field is unequivocally directed toward the development of precision strategies. The cornerstone of this approach is achieving precise spatiotemporal control over drug delivery and action, which is fundamentally dependent on guidance from a robust biomarker framework for accurate patient stratification. Future breakthroughs will depend on the deep integration of targeted delivery technologies with this biomarker system.

To overcome the drawback of the nonspecific systemic distribution exhibited by current agents, the development of delivery systems capable of precisely targeting diseased cardiac cells is paramount. Nanoparticles decorated with ligands for cardiomyocyte-specific receptors enable passive targeting (e.g., the EPR effect), active targeting, and intelligent drug release in response to the pathological microenvironment (e.g., pH and enzymes), ensuring drug enrichment and localized release at the cardiac site 255, 256. Designing prodrugs that are inactive in the systemic circulation but are selectively activated by enzymes overexpressed in diseased tissues (e.g., cathepsins and matrix metalloproteinases) allows for the localized release of active copper-modulating agents, thereby minimizing off-target effects 257. Utilizing tissue-specific promoters to drive the localized expression of copper transporters or chelating proteins within specific organs (e.g., the heart) allows for fine-tuned modulation of local copper homeostasis without disrupting systemic balance 258.

The clinical translation of the aforementioned precision strategies urgently requires a biomarker system capable of dynamically and specifically reflecting disruptions in copper homeostasis and their pathological consequences. Serum CP, as the primary copper transport protein, reflects systemic copper status, inflammation, and oxidative stress. Its elevation in conditions such as HF signals an underlying copper and redox imbalance 259, 260. Non-ceruloplasmin-bound copper (NCC) represents the redox-active “free” copper fraction, which is a key driver of oxidative injury and cuproptosis. An increase in NCC may indicate an acute pathological risk, even when total copper levels are within the normal range 261. Soluble SLC31A1 reflects the activity of cellular copper uptake via CTR1. Elevated levels may indicate increased tissue susceptibility to copper-mediated damage, particularly when cellular compensatory mechanisms, such as copper buffering or efflux, are impaired 262. The high expression of copper-related genes, such as FDX1 and SLC31A1, in metabolic organs, such as the liver and intestine, poses a challenge in interpreting their specific roles in cardiovascular pathology, underscoring the need for tissue-specific models 263. Integrating these copper-specific markers (e.g., CP and NCC) with established cardiac biomarkers, such as BNP and troponin, can help identify a “high copper risk” patient subgroup, thereby informing personalized treatment decisions 264. Furthermore, dynamic monitoring of these biomarkers holds promise for elucidating the role of cuproptosis in disease progression, particularly in patients refractory to standard therapies.

The key to advancing the field of copper-targeted therapy lies in the systematic coupling of innovative spatially precise delivery technologies with a dynamic multi-level biomarker framework. Only through such an integrated strategy can the modulation of copper homeostasis be transformed from a blunt systemic intervention into a truly precise and personalized therapeutic approach for treating cardiovascular diseases. Future research must focus on facilitating the clinical validation and translation of these technologies, ultimately enabling the safe and effective use of copper for the prevention and treatment of cardiovascular diseases.

## Conclusions

Copper homeostasis is a fundamental pillar in sustaining cardiovascular health, and its dysregulation is a pivotal driver of diverse cardiovascular pathologies. This review systematically unravels the intricate interplay between copper metabolism and cuproptosis, a unique copper-dependent RCD pathway, in the onset and progression of cardiovascular diseases. From myocardial I/R injury to DCM, atherosclerosis, and HF, cuproptosis mediates pathogenic processes through mechanisms including mitochondrial dysfunction, proteotoxic stress, GSH depletion, and lipid metabolism disruption.

Notably, the dual nature of copper imbalance—both deficiency and excess—contributes to cardiovascular damage via distinct yet interconnected pathways. Copper deficiency impairs mitochondrial respiration, antioxidant defense, and vascular structural integrity, whereas copper overload triggers oxidative stress, inflammatory responses, and aberrant activation of cell death signaling. Modulating copper homeostasis, either through chelation to mitigate overload or supplementation to correct deficiency, has demonstrated therapeutic potential for alleviating tissue damage and improving cardiac function in preclinical models.

The translational value of targeting cuproptosis and copper metabolism lies in the identification of key regulatory molecules (e.g., FDX1, DLAT, ATP7A/B) and potential biomarkers (e.g., serum CP and NCC) that could guide clinical decision-making. However, current challenges, such as the lack of tissue-specific targeting of copper-modulating agents and interspecies differences in preclinical models, necessitate further refinement of therapeutic strategies.

Future research on cuproptosis in cardiovascular diseases should advance along these critical paths. First, elucidating the cell-type-specific sensitivities and regulatory mechanisms of cuproptosis in major cardiac cells, including cardiomyocytes, ECs, and fibroblasts, is essential. Furthermore, understanding how copper homeostasis is dysregulated in comorbid conditions (e.g., diabetes and HF) will provide a more comprehensive pathophysiological perspective. Translational efforts should focus on developing cardiac-specific modulators of copper homeostasis, such as small-molecule inhibitors targeting the copper transporter CTR1 in cardiomyocytes. Concurrently, screening for clinically applicable biomarkers—such as serum NCC levels or cardiomyocyte-enriched microRNAs—is crucial for patient stratification and monitoring.

## Figures and Tables

**Table 1:** Molecular intersections and distinctions between cuproptosis and other regulated cell-death pathways

Cell death pathway	Primary trigger	Morphological features	Biochemical characteristics	Core execution mechanism	Key molecules/ hallmarks	Key genes	Ref.
Cuproptosis	Excess copper ions	Mitochondrial shrinkage and plasma membrane rupture	Cu^2+^ binds to lipoacylated DLAT and induces disulphide bond-dependent aggregation of lipoylated DLAT	Copper-binding to lipoylated TCA cycle proteins, leading to protein aggregation and proteotoxic stress.	Copper, FDX1, and lipoylated DLAT	FDX1	265
Apoptosis	Death receptor signaling, DNA damage, and cellular stress	Apoptotic bodies' chromatin condensation, DNA fragmentation, and cell shrinkage	DNA fragmentation, and the dismantling of cellular components	Caspase activation, DNA fragmentation, and the dismantling of cellular components.	Caspases, BCL-2 family, and cytochrome c	BCL-2, Bak, and caspases	266
Ferroptosis	Iron-dependent generation of lipid peroxidation	Small mitochondria and elevated mitochondrial membrane density.	Decreased expression of GSH and GPX4, increased divalent iron and lipid peroxidation	Inactivation of GPX4, leading to lethal accumulation of lipid peroxides.	Iron, lipid peroxides, and GPX4	GPX4	267
Pyroptosis	Pathogen *Shigella* infection	Cell swelling, plasma membrane leakage, and chromatin condensation	Caspase-1 activation dependent, GSDMD cleavage and inflammatory factor release	Pore formation in the plasma membrane, causing inflammatory cell lysis.	Gasdermin D, inflammasome, and IL-1β	GSDMD, NLRP 3, and caspases-1/4/5	268
Necroptosis	Tumor necrosis factor receptor signaling and caspase-8 inhibition	Disintegration of plasma membrane, swelling of organelles, and spillage of cellular contents	Activation of RIPK1, RIPK4, and MLKL and a decrease in ATP levels	RIPK1/RIPK3/MLKL-mediated plasma membrane rupture.	RIPK3 and phosphorylated MLKL	RIPK3 and MLKL	269

**Table 2:** Key cuproptosis molecular changes in cardiovascular diseases

Disease/condition	FDX1	DLAT	SLC31A1 (CTR1)	ATP7A/B expression	Key pathological outcome	Key evidence and proposed mechanisms	Ref.
Myocardial I/R injury	↑	↑	↑	-	Increased cardiomyocyte cuproptosis; larger infarct	Reperfusion triggers intense oxidative stress and mitochondrial dysfunction, leading to coordinated upregulation of copper uptake and cuproptosis execution, resulting in secondary cardiomyocyte death.	17
Doxorubicin induced cardiotoxicity	↓	↑	↑	ATP7B ↓	Mitochondrial injury and HF progression	Doxorubicin directly targets mitochondria, inducing severe oxidative stress and clearly activating the FDX1/DLAT-mediated cuproptosis pathway.	74
Diabetic cardiomyopathy	↑	↑	↑	↓	Impaired contractility and myocardial fibrosis	Hyperglycemia and metabolic disorders cause persistent mitochondrial stress, creating a pro-cuproptotic environment. The coordinated upregulation accelerates cardiomyocyte loss and fibrosis.	169
Sepsis-induced cardiomyopathy	↑	-	↑			Systemic inflammation and oxidative stress can activate FDX1 in the heart, leading to myocardial injury and functional impairment.	270
Atherosclerotic cardiovascular disease	↑	-	↑	ATP7A↑	Endothelial dysfunction	In vascular endothelial and smooth muscle cells, inflammation and oxidative stress can activate cuproptosis, promoting cell death.	187
Heart failure	↓	↑	↓	ATP7A↑	Progressive failure of the pump function	Heart failure and fibrosis of the heart were more obvious, suggesting the existence of a large number of cardiomyocyte loss.	100
Aortic aneurysm	↓	↑	-	-	Elastic fiber rupture, loss of smooth muscle cells	Cuproptosis is activated in vascular smooth muscle cells (VSMCs), leading to VSMC loss, which weakens the aortic wall.	271
Hypertension	↓	↑	↑	ATP7A↓	Cardiac hypertrophy and myocardial fibrosis	Pressure overload leads to hypertrophy and mitochondrial dysfunction	12

**Table 3:** Characteristics of conventional cardiovascular drugs and copper metabolism-related drugs

Class of agent	Representative drugs	Core mechanism	Indications	Key adverse effects	Ref.
Conventional drugs	Metoprolol (β-blocker)	Blocks β-adrenergic receptors to inhibit sympathetic nervous system excitation	Hypertension, heart failure, and arrhythmia	Bradycardia, fatigue, and bronchospasm	272
Conventional drugs	Enalapril (ACE inhibitor)	Inhibits angiotensin-converting enzyme, reducing the production of the potent vasoconstrictor angiotensin II	Hypertension, heart failure, cardiac protection after myocardial infarction, and diabetic nephropathy	Dry cough, angioedema, hyperkaliemia, and kidney damage	273
Conventional drugs	Losartan/valsartan (ARB)	Blocks the binding of angiotensin II to its receptor	Hypertension, heart failure, and diabetic nephropathy	Hyperkaliemia and kidney damage	274
Conventional drugs	Atorvastatin (statin)	Inhibits HMG-CoA reductase and lowers LDL cholesterol	Atherosclerosis, atherosclerotic cardiovascular disease (ASCVD)	Muscle soreness or myopathy, elevated liver enzymes, and risk of new-onset diabetes	275
Copper metabolism-Targeted agents	TTM (trientine tetrahydrochloride) and TETA (trientine)	Chelates labile Cu⁺ and reduces bioavailable copper	Copper metabolic disorders	Copper deficiency, bone marrow suppression, and kidney damage	276
Copper metabolism-Targeted agents	Elesclomol (copper ionophore, repurposed)	Binds to extracellular copper ions (Cu²⁺) to form complexes that enter cells	Cancer treatment	Oxidative stress-related toxicity and mitochondrial dysfunction	58

**Table 4:** Copper chelators for use in clinical trials

Conditions	Phases	Primary Objective	Results	Enrolled	ClinicalTrials.gov identifier	Status	Organizing Location
Wilson Disease		The period of the day best correlated with 24 h urinary copper excretion		30	NCT06430359	Recruiting	France
Head and Neck Cancer	II	The safety and efficacy of penicillamine (a common copper chelator)		10	NCT06103617	Recruiting	China
Wilson Disease		Assess copper parameters in participants with Wilson disease		64	NCT02763215	Completed	United States/ Austria/Germany/ Poland/United Kingdom
Wilson Disease		The clinical efficacy and safety of trientine		48	NCT03299829	Completed	Taiwan
Idiopathic Pulmonary Fibrosis	I/II	The safety of the administration of a copper chelating agent, tetrathiomolybdate	The primary endpoint is safety with secondary endpoints including change in pulmonary function, exercise capacity, and quality of life	23	NCT00189176	Completed	United States
Psoriasis Vulgaris	II	The safety and efficacy of Tetrathiomolybdate in psoriasis therapy		10	NCT00113542	Completed	United States
Wilson Disease	I/II	the safety of single IV doses of UX701		82	NCT04884815	Active, not recruiting	United States/Canada/ Portugal/Spain/ United Kingdom
Wilson Disease	Not Applicable	A single daily treatment with trientine is as effective or better than a patient's current maintenance therapy	The primary endpoint is the demonstration of equivalence to a patient's prior therapy. Secondary endpoints include: 1) demonstration of stability or improvement in parameters of copper metabolism; 2) improvement in adherence to therapy; and 3) no progression of liver disease	8	NCT01472874	Completed	United States
Epithelial Ovarian Cancer	I/II	copper chelator in conjunction with cytotoxic agents to conquer platinum-resistance		18	NCT03480750	Completed	Taiwan
